# Pharmacokinetic Study and Bioavailability of a Novel Synthetic Trioxane Antimalarial Compound 97/63 in Rats

**DOI:** 10.1155/2014/759392

**Published:** 2014-09-11

**Authors:** Hari Narayan Kushwaha, Neel Kamal Mohan, Ashok Kumar Sharma, Shio Kumar Singh

**Affiliations:** ^1^Pharmacokinetics and Metabolism Division, CSIR-Central Drug Research Institute, Lucknow 226001, India; ^2^Medicinal and Process Chemistry Division, CSIR-Central Drug Research Institute, Lucknow 226001, India

## Abstract

Single dose pharmacokinetics study of 97/63 (IND191710, 2004), a trioxane antimalarial developed by Central Drug Research Institute, Lucknow, India, was studied in rats following intravenous and oral administration. Serum samples were analysed by HPLC-UV assay. Separation was achieved on a RP-18 column attached with a guard using acetonitrile : phosphate buffer (70 : 30% v/v) with UV detector at wavelength 244 nm. Serum samples were extracted with *n*-hexane. Two-compartment model without lag time and first-order elimination rate was considered to be the best fit to explain the generated oral and intravenous data. Method was sensitive with limit of quantification of 10 ng mL^−1^. Recovery was >74%. Terminal half-life and area under curve (AUC) after administering single oral (72 mg kg^−1^) and intravenous (18 mg kg^−1^) doses were 10.61 h, 10.57 h, and 1268.97 ng h mL^−1^, 2025.75 ng h mL^−1^, respectively. After oral dose, 97/63 was rapidly absorbed attaining maximum concentration 229.24 ng mL^−1^ at 1 h. Bioavailability of 97/63 was ~16%. The lower bioavailability of drug may be due to poor solubility and first-pass metabolism and can be improved by prodrug formation of 97/63.

## 1. Introduction

World Health Organization (WHO) has recommended artemisinin class of compounds as promising novel antimalarials for further clinical development [1]. Several semisynthetic derivatives of artemisinin, the active ingredient of Chinese herb “qing hao” (*Artemisia annua*) used traditionally for treating fevers, have been used increasingly over past two decades. Discovery of artemisinin, as the active principle of Chinese traditional drug* Artemisia annua*, has opened new possibilities in malaria chemotherapy. Artemisinin and its more potent derivatives (artemether, artemotil, and artesunic acid, etc.) are highly active against both chloroquine-sensitive and resistant malaria. These drugs are fast acting and are well suited for treatment of cerebral malaria caused by* P. falciparum* (drug sensitive as well as drug resistant species). Peroxide group, present in form of 1,2,4-trioxane, is essential for antimalarial activity of these drugs. The endoperoxide sesquiterpene lactone moiety of this class of compounds is found to be indispensable for the erythorocytic schizontocidal activity and reacts with intraparasitic heme and forms free radicals. These free radicals appear to damage intracellular targets and perform their antimalarial activity [2–4]. However, the existing artemisinin class of drugs is to be improved regarding efficacy, neurotoxicity, stability, and pharmacokinetic (PK) behaviors. Synthesis of a large number of structurally simple trioxanes have been reported, several of which have shown promising* in vivo* antimalarial activity [5].

**Figure 1:**
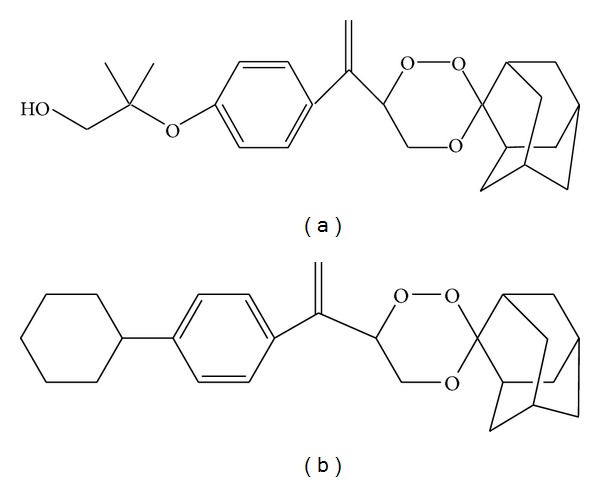
Chemical structure of parent compound 97/63 (a) and IS (b).

Central Drug Research Institute (CDRI) developed a promising antimalarial compound, 97/63 (Racemate) (Figure 1(a)), in its drug discovery program. 97/63 showed good potency against malarial parasites,* in vitro* as well as* in vivo *[6]. It possesses a 1,2,4-trioxane nucleus similar to endoperoxide lactone of artemisinins, essential for antimalarial activity [7–9]. Several synthetic trioxanes, simplified analogs of artemisnin retaining the crucial endoperoxide bridge, have been reported but only a few have entered successfully in clinical trials till date [6]. Firstly, 97/63 was synthesized which showed promising antimalarial activity but, due to its poor bioavailabilty, it was resynthesized as a hemisuccinate derivative and coded as 97/78 which is currently into clinical trials Phase I. The 1,2,4-trioxane nucleus present in antimalarial 97/63 is responsible for its pharmacological activity [7–11]. In continuation of drug development program after successful completion of pharmacological activities, the preclinical development was undertaken. So, the present study describes pharmacokinetic behavior of 97/63 after single oral and intravenous dose administration in male rats. Characterization of preclinical pharmacokinetic studies in animal models is required for toxicological and preclinical studies and also for extrapolation of pharmacokinetics and pharmacodynamics in humans [10].

Analytical method was developed and validated in terms of sensitivity, specificity, accuracy, precision, and recovery. The study samples were analyzed using validated HPLC-UV assay developed for quantification of 97/63 in rat serum.

## 2. Materials and Methods

### 2.1. Chemicals and Reagents

Pure reference standards (purity >99%) of 97/63 and 99/411 (Figure 1(b)) used as internal standard (IS) were obtained from Medicinal and Process Chemistry Division, CDRI, Lucknow, India. Acetonitrile, HPLC grade, was purchased from Thomas Baker (Chemicals) Limited (Mumbai, India). Analytical grade potassium dihydrogen orthophosphate for buffer preparation was supplied by SD Fine-Chem Limited, Mumbai, India. Orthophosphoric acid (analytical grade) for pH adjustment of the buffer was purchased from Qualigens Fine Chemicals, Mumbai, India. For sample extraction,* n*-hexane was obtained from SD Fine-Chem Limited, Mumbai, India. Ultrapure water of 18.2 MΩ cm was obtained from a Milli-Q PLUS PF system. Drug-free pool of serum was obtained from male rats (Sprague-Dawley, SD) which were obtained from Laboratory Animal Services Division, CDRI. The separated serum was stored at −60°C till use. Animals were cared for in accordance with the guidelines laid by local ethical committee for animal experimentation and the principles of the guide for care and use of laboratory animals were followed (Department of Health Education and Welfare, number [NIH] 85-32).

### 2.2. HPLC-UV Instrumentation and Analytical Conditions

HPLC system consisted of a pump (LC-10ATvp with SCL 10Avp system controller, Shimadzu, Japan) with quaternary flow control valve system (FCV-10ALvp) and a degasser (DGU-14A) to pump the mobile phase. The detection of 97/63 and IS was performed using SPD-10A UV-VIS detector at 244 nm. SIL-10ADvp Shimadzu autoinjector was used to inject samples. Chromatographic separation was performed isocratically on Spheri-5, RP-18 column (100 × 4.6 mm i.d. 5 mm) (Pierce Chemicals Co., Rockford, IL, USA) coupled with a guard column (30 × 4.6 mm i.d., 5 mm) packed with the same material. The mobile phase composed of acetonitrile : phosphate buffer (10 mM, pH 4.0) (70 : 30% v/v) at flow rate 1 mL min^−1^. Phosphate buffer was prepared by dissolving 1.36 g of potassium dihydrogen orthophosphate in one liter triple-distilled water and its pH 4.0 was adjusted with 40% orthophosphoric acid. Phosphate buffer was filtered with 0.22 *μ*m cellulose membrane under vacuum and degassed for 20 min in sonicator (Bransonic cleaning Co., USA) before use. Data was analysed using CLASS-VP software (Shimadzu, Japan) running on Compaq PC. HPLC system was equilibrated for approximately 30 min at flow rate 1 mL min^−1^ before the commencement of analysis. The chromatographic run time was 10 min and injection volume was optimized to 20 *μ*L. A vortex-mixer (Thermolyne, USA), Model SVC-220H speed vac concentrator (Savant, NY, USA) and Model K130 centrifuge (BHG Hermle), was used for sample preparation. Serum samples were stored at −60°C in Ultra Freeze U41085, Ultra Low Freezer (New Brunswick Scientific, USA).

### 2.3. Stock and Standard Solutions

Stock solution (1 mg mL^−1^) of 97/63 and IS was prepared by dissolving separately 10 mg of compounds in 10 mL acetonitrile. Working stock solutions of strengths 10 *μ*g mL^−1^ and 50 *μ*g mL^−1^ were prepared by transferring 100 *μ*L and 500 *μ*L of stock solution into volumetric flask and final volume was made up to 10 mL with acetonitrile. These solutions were serially diluted in mobile phase by adding 10 *μ*L mL^−1^ of working stocks to get 10, 25, 50, 100, 250, and 500 ng mL^−1^ concentrations. Spiking solution of IS was prepared by diluting 10 *μ*L of mother stock with 1990 *μ*L of acetonitrile to yield solution of 5 *μ*g/mL. These analytical standards were used to determine HPLC system reproducibility and absolute recovery for 97/63 from biomatrix used. All solutions were stored at 4°C.

### 2.4. Calibration Curve

Calibration standards (CS) samples of 97/63 from 10–500 ng mL^−1^ in rat serum were prepared by adding appropriate volumes of working stocks in 200 *μ*L of pooled rat serum so that the volume ratio of organic phase added was less than 2.5%. CS and quality control (QC) standards were stored at −60°C until analysis. QC samples at low (10 ng mL^−1^), medium (100 ng mL^−1^), and high (500 ng mL^−1^) concentrations were used for the method validation program. Prior to HPLC analysis these CS and QC samples were processed according to method outlined in the following paragraph.

### 2.5. Sample Preparation

CS, QC, and test samples were prepared separately using a simple and efficient two-step liquid-liquid extraction process using* n*-hexane. To the blank or spiked serum (200 *μ*L), 10 *μ*L of IS was added to each sample to get 250 ng/mL of 99/411 and 3 mL of* n*-hexane was added. This mixture was vortexed for 3 min followed by centrifugation at 2200 ×g, at 4°C for 10 min. The organic layer was transferred by snap freezing the aqueous layer in liquid nitrogen into a clean tube and evaporated to dryness in speed vac concentrator. The method was repeated and reextraction was carried out in similar manner. The dry residue was reconstituted in 200 *μ*L mobile phase and injected for analysis.

### 2.6. Method Development and Validation

Method development involved use of different columns (Cyano and RP-18) to check noninterference in the region of compound of interest with endogenous substances, drug metabolite, and so forth in the determination of concentration [12]. Use of different buffers with different pH range (pH 4–7) was checked in different compositions of various organic solvents. HPLC system reproducibility was checked with five replicate injections of each analytical standard. HPLC-UV method was validated for five days at three different concentrations (10, 100, and 500 ng mL^−1^ as low, medium, and high QC resp.) in five replicates. The method developed was validated in terms of HPLC system sensitivity, specificity, linearity, absolute recovery, accuracy, precision, freeze-thaw (F-T) cycle stability, long-term stability at −60°C, dry residual stability, and autosampler stability. The lower limit of detection (LOD) for 97/63 was the quantity in the serum after the sample cleanup corresponding to three times the baseline noise (S/N > 3). The limit of quantitation (LOQ) is the concentration of sample that can be quantified with less than 20% variation in precision [13].

Matrix effect between analytes, IS, and coeluents from biometrices might probably exist. So matrix effect was evaluated by peak area ratio of the analytes dissolved in the supernatant of processed blank serum to that of analytical standard of same concentration. Five samples of each set at QC of 97/63 were evaluated. Moreover highly sensitive methods could be affected by carryover, visible in LC-UV detection. In this study carryover was evaluated by analyzing the mobile phase after plasma samples with the highest analyte concentration 2000 ng mL^−1^.

Linearity for CS (*n* = 6) in five replicates for five days was assessed by subjecting the peak area of spiked concentrations to linear regression analysis (*y* = *mx* + *c*) with 1/*x* ^2^ weighing factor showed the best fit for the calibration curve. The choice of proper calibration method depends on the residuals obtained and the coefficient of correlation [14, 15].

The recovery of 97/63 prior to analysis was determined at QC samples of low (10 ng mL^−1^), medium (100 ng mL^−1^), and high (500 ng mL^−1^) concentrations. The extraction recoveries at three QC levels were determined by comparing peak areas obtained from plasma samples with those found by direct injection of standard solutions prepared in mobile phase of same concentrations. Recovery of IS was also calculated.

Accuracy was determined by injection of calibration standards and QC standards in five replicates on five different days (*n* = 75, five each of low, medium, and high concentration). The interday and intraday accuracy was determined by calculating % bias from the theoretical concentration using equation (% bias = (observed concentration − nominal concentration)/nominal concentration × 100) of quality control samples. The interday and intraday precision was determined by subjecting the data to one-way analysis of variance (ANOVA) in terms of relative standard deviation (% RSD).

### 2.7. Stability Studies

Long-term stability of 97/63 was carried by preparing QC samples at low, medium, and high concentrations in three replicates for four different days and stored at −60°C. These sets of samples were analyzed after 0, 7, 15, and 30 days of storage and their concentrations were read from the respective calibration standard curve. The results are expressed as % deviation from 0 days concentration.

F-T stability of 97/63 in serum samples was determined by preparing QC of strengths low (10 ng mL^−1^), medium (100 ng mL^−1^), and high (500 ng mL^−1^) in three replicates for four different days. One set of three concentrations was analyzed on day of preparation (no F-T cycle) before storing the remaining sets at −60°C. Other sets were analyzed after 1, 2, and 3 F-T cycles. Thawing was achieved by keeping the sealed tubes at room temperatures for 30 min. The results are expressed as % deviation with initial concentration.

The effect of storage on dry residue stability was determined at QC 10 ng mL^−1^ (low), 100 ng mL^−1^ (medium), and 500 ng mL^−1^ (high) concentration levels. All the samples (three replicates of three concentrations × 4 sets) were prepared and dry residue was stored in glass tubes sealed with aluminum foil and then with parafilm. One set in triplicate was reconstituted on day 1 with mobile phase and analyzed on the same day (without storage). The remaining sets were similarly analyzed on days 2, 4, and 8 and results are expressed as % deviation.

After sample preparation, stability of 97/63 in the autoinjector was evaluated for 4 h; the maximum duration for which the sample may have to wait in the autoinjector tray for pending analysis and results are expressed in % deviation with initial injection (*t* = 0 *h*).

### 2.8. Subjects

Male Sprague-Dawley (SD) rats weighing 250 ± 25 g for this study were obtained from institutional Laboratory Animal Division, CDRI, Lucknow, prior to study. Rats were given standard pellets for feeding and filtered water was given* ad libitum* and they were housed in well ventilated cages which were kept at room temperature on a regular 12-h light-dark cycle. Animals were kept in accordance with the guidelines of local ethical committee (Ethical approval 58/08/PKM/IAEC) for animal experimentation and principles of the guide for care and use of laboratory animals were followed (Department of Health Education and Welfare, number [NIH] 85-32). Oral and IV study was carried out in two sets (I and II). Each set contained two groups, that is, groups A and B. PK was carried out by dosing 97/63 in two groups (A and B) containing four rats in each group for sparse sampling.

### 2.9. Pharmacokinetic Study and Statistical Analysis

Young and healthy male SD rats weighing 250 ± 25 g (obtained from Laboratory Animal Division of CDRI) were housed in well ventilated cages and kept at room temperature 25 ± 2°C on a regular 12-h light-dark cycle. Animals were cared for in accordance with the principles of guide for care and use of laboratory animals. Animals in oral study group were fasted overnight to minimize the effects of food on pharmacokinetic profile of 97/63. Intravenous dose was administered as a single bolus injection through tail vein using a 26 G needle after dilating the tail vein [16]. The oral dose was administered using a 2 mL syringe and feeding needle. After intravenous and oral dose administration, rats were also monitored for physiological behavior.

An oral dose of 72 mg kg^−1^ and intravenous dose of 18 mg kg^−1^ as suggested by the efficacy studies were, therefore, used in the study. The intravenous formulation for single dose, 18 mg kg^−1^, was a solution of 97/63 in DMSO. The oral formulation for single dose, 72 mg kg^−1^, was also a solution of 97/63 in DMSO. The formulations were subjected to quality control (QC) checks to ensure their strength and content uniformity prior to use. The strength of formulation for intravenous and oral administration was 18 mg mL^−1^ and 36 mg mL^−1^. Compound 97/63 formulated in DMSO was administered to overnight fasted rats by oral and intravenous route. The dose volume factor for intravenous administration was 1 mL kg^−1^, with 250 g rat receiving 0.25 mL, and for oral administration it was 2 mL kg^−1^, with 250 g rat receiving 0.5 mL of formulation. Sparse sampling approach was used to collect blood samples by cardiac puncture. The total volume of blood withdrawn within 24 hours was less than 5% of blood volume. Blood samples were collected in glass tubes at 0.083, 0.25, 0.5, 0.75, 1, 1.5, 2, 3, 4, 6, 8, 10, 18, 24, 48, and 60 h after oral dose. Blood samples were stored at room temperature for 30 min to separate the serum. Serum was separated by centrifugation at 1500 ×g for 10 min and stored at –60°C until analysis. Analysis was performed within 30 days of sample collection.

Curve fitting and estimation of pharmacokinetic parameters of 97/63 were carried out using WinNonlin (5.1) software program (Pharsight Corp, USA). The data were tested using Gauss-Newton algorithm, with noncompartment, one compartment, and two-compartment models after applying different weighting factor to the observed values of the concentrations. Decision on the suitability of a particular compartment model in explaining the data was undertaken based on the Akaike or Schwarz information criterion, correlation coefficient, and scatter of residuals.

Data are reported as means ± SD and statistical analysis was performed using one way analysis of variance (ANOVA) at 95% confidence interval (CI).

## 3. Results

The successful analysis of analytes in biological fluids using HPLC relies on the optimization of sample preparation, chromatographic separation, and postcolumn detection. Each of these steps was carefully optimized for developing sensitive, selective, and reproducible assay of 97/63 in rat serum.

### 3.1. Method Validation

The chromatographic conditions were modified to get better selectivity and sensitivity. Thus, molarity and pH of buffer and type of columns were optimized. The effect of molar strength and nature of buffers in mobile phase on peak shape of 97/63 was studied [17]. Buffers like ammonium acetate and potassium dihydrogen orthophosphate were used for the preparation of mobile phase. The use of buffer is particularly important in separating polar compounds on reversed phase column, since a properly chosen pH can alter the retention time of the eluting peaks. The absence of buffer salt resulted in increased retention time, peak broadening, and hence lower sensitivity. Appropriate ratio of buffer and organic solvent leads to better separation with decreased retention time, sharpening of peak, and adequate separation from the inherent serum components. Phosphate buffer showed good peak shape and retention time at pH 4. Different columns (Cyano and RP-18) of varying length were used to check the peak shape and retention time. The isocratic elution with 70 : 30 acetonitrile : phosphate buffer at 1 mL min^−1^ on Spheri-5, RP-18 column showed no interference with any endogenous impurity. The retention time of 97/63 and IS was 6.38 ± 0.25 and 8.21 ± 0.07 min, respectively. The new assay developed for 97/63 was found to be specific. No significant suppression between 97/63 and IS was observed throughout the analysis. 99/411 was selected as IS due to structural analogue and similar physicochemical properties. The HPLC system reproducibility was checked with five replicate injections of each analytical standard and the % bias and % RSD were between 12.51 to −12.36 at all concentrations. The variations in the peak areas of 97/63 were maximal at 10 ng mL^−1^ (3.9%) and were less than 2.0% at all other concentration levels indicating that the system yields reproducible data. The results showed that the variations were within the acceptable limit of *P* > 0.05 in one-way ANOVA.

**Figure 2:**
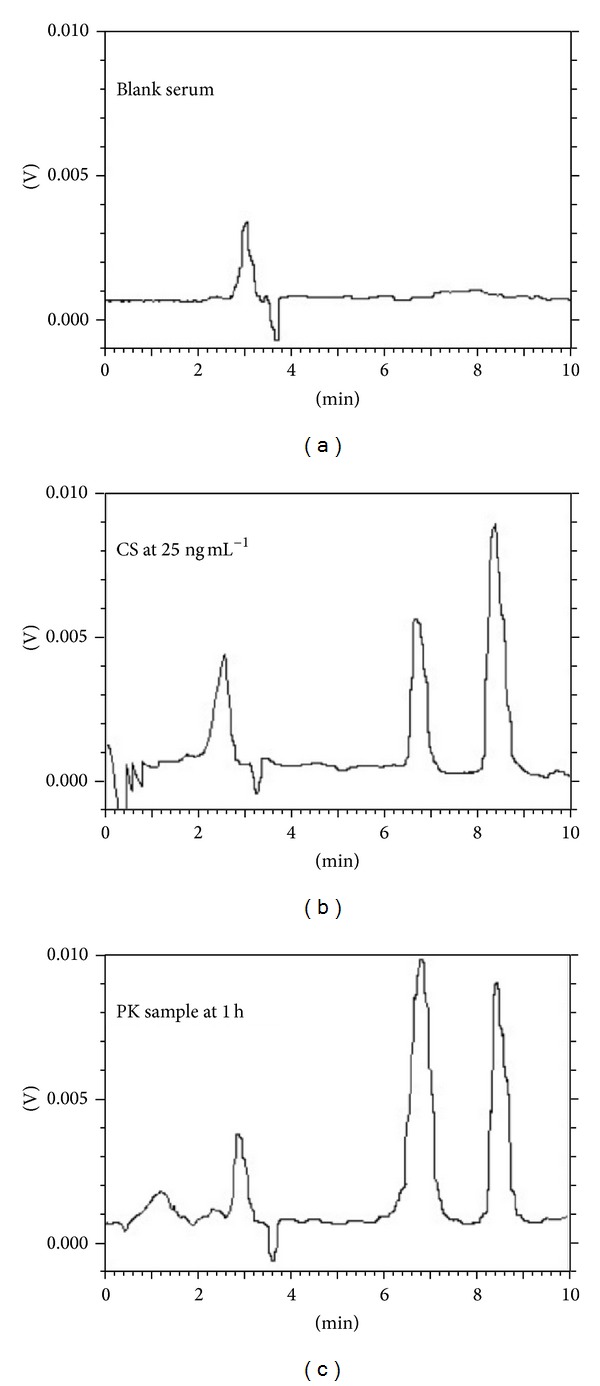
Representative chromatograms of (a) blank serum sample, (b) calibration standard at 25 ng mL^−1^ concentration, and (c) test sample of 1 h after oral (72 mg kg^−1^) dose.

Sample clean-up techniques were also optimized to get rid of interfering endogenous substances without sacrificing the recovery of 97/63 and IS. A proper selection of solvent was essential for yielding maximum recoveries. Extraction solvents like* n*-hexane, diethyl ether, ethyl acetate, and different composition of isopropyl alcohol in* n*-hexane were used. The best results were obtained with* n*-hexane without compromising recoveries. The HPLC chromatogram (Figure 2) shows no endogenous peak interferes with peaks of interest. The chromatographic conditions and extraction procedure yielded a clean chromatogram for analyte. The recovery of 97/63 and IS from spiked serum sample was calculated by comparing the peak area with those obtained from mobile phase standards. The absolute recoveries of 97/63 over the range 10–500 ng mL^−1^ from serum are summarized in Table 1.

No significant suppression for 97/63 by matrices was observed. No detectable carryover occurred in mobile phase runs after plasma samples determinations.

The peak area of 97/63 in rat serum varied linearly with concentration over the range 10–500 ng mL^−1^. The calibration model was selected on individual calibration data by linear regression with or without intercept and weighing factor. The linear regression equation was *y* = 5.228*x* − 3.869 and the regression coefficient for standard calibration curve was *R* ^2^ = 0.999. LOD and LOQ for 97/63 were 5 and 10 ng mL^−1^ respectively. The % accuracy and % RSD at three concentrations in five replicates for 97/63 are presented in Table 2 for complete validation in rat serum. The result showed that the analytical method was accurate as the intraday and interday variations (13.42 to −14.00) were within the acceptable limit ±20% for low and ±15% for all other concentrations. Similarly the % RSD for different QC samples was between 14.8 and 4.71, well within the acceptable limit.

### 3.2. Stability Studies

**Figure 3:**
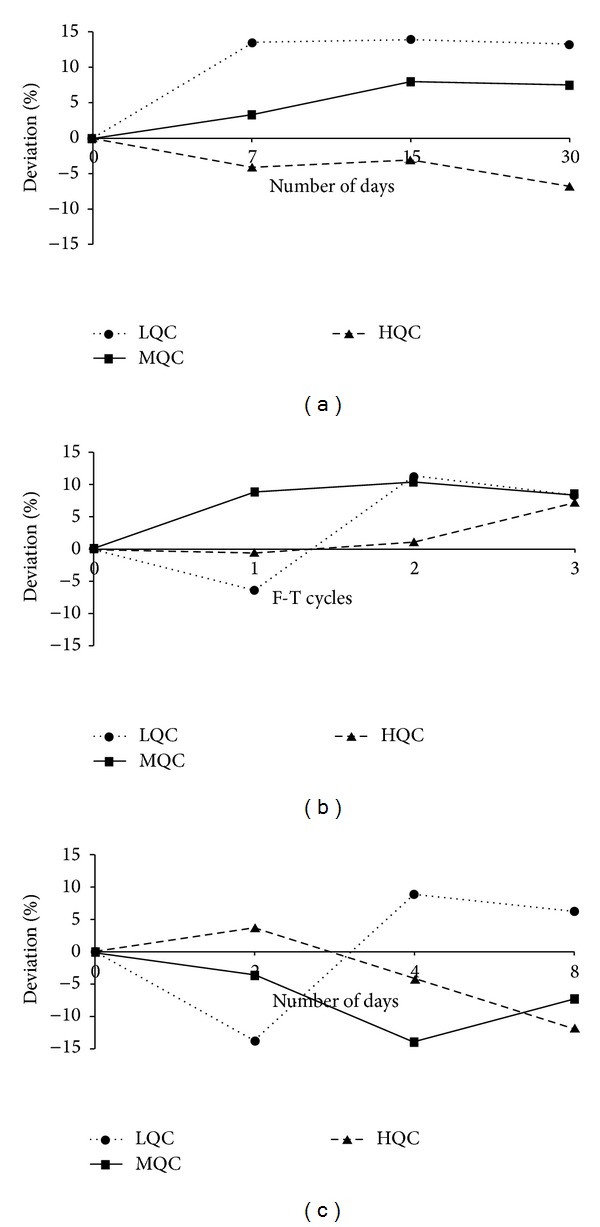
Stability studies: (a) long-term stability, (b) freeze-thaw stability, and (c) dry residual stability.

97/63 was found to be stable over a period of 30 days in normal serum when stored at −60°C (Figure 3(a)). The analyte was found to be stable after three F-T cycles (Figure 3(b)) and the dry residue after extraction was also found to be stable over a period of 8 days when stored at −60°C (Figure 3(c)). The percent deviation calculated was within ±20% at low concentration and within ±15% for all other concentrations. The reinjection reproducibility was established to determine if an analytical run could be reanalyzed in case of unexpected delay in analysis. The same set of QC samples was repeated after one analysis with 4 h gap in between the samples which were stored at 4°C and in all cases the deviations were less than 10%.

### 3.3. Pharmacokinetic Study

**Figure 4:**
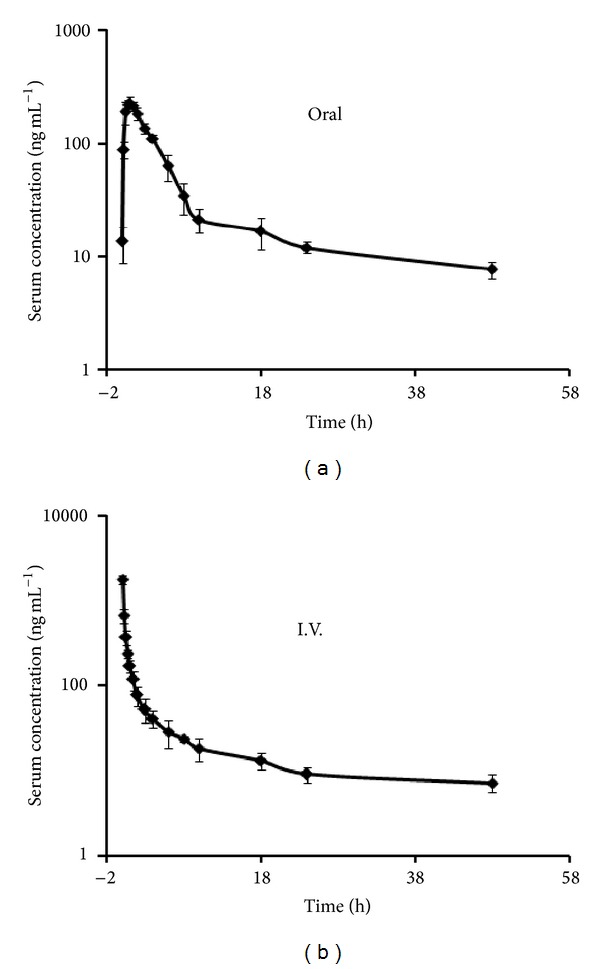
Log-linear scale of mean serum concentration (ng mL^−1^) versus time profile of 97/63 following (a) oral at 72 mg kg^−1^ and (b) intravenous at 18 mg kg^−1^ dose administration in male rats (mean ± SD, *N* = 4).

Following oral dose administration in male rats, the levels of 97/63 showed biexponential decay up to 48 h. A two-compartment 1st-order pharmacokinetic model without lag time and first-order elimination rate was considered to be the best fit to explain the generated data. Mean serum concentration-time profile of 97/63 after single oral dose of 97/63 in male rats is shown in Figure 4(a).  Table 3 summarizes the pharmacokinetic parameters of 97/63 after oral administration at 72 mg kg^−1^ to male rats. The *C*_max⁡_, *T*_max⁡_, and AUC_0–*∞*_ were found to be 229.24 ± 64.26 ng mL^−1^, 1 ± 0.7 h, and 1268.97 ± 27.04 ng*·*h mL^−1^, respectively. The distribution (*t*_1/2_ *α*) and elimination (*t*_1/2_ *β*) half-life were found to be 0.84 ± 0.14 and 10.61 ± 0.2 h, respectively. 97/63 was found to be rapidly absorbed without showing any lag time for absorption. Peak levels were attained at one hour (*T*_max⁡_ 1 h).

Serum concentration-time profile of 97/63 in male rats after intravenous administration at 18 mg kg^−1^ dose has been depicted in Figure 4(b). The initial concentration (*C*_0_) and AUC_0–*∞*_ were found to be 1799.99 ± 330.24 ng mL^−1^ and 2025.75 ± 574.3 ng*·*h mL^−1^, respectively. The distribution and elimination half-life were found to be 0.45 ± 0.08 and 10.57 ± 0.16 h, respectively. The detailed pharmacokinetic parameters of postintravenous dose are listed in Table 3.

The absolute bioavailability of compound 97/63 was calculated to be 15.66%. Poor bioavailability may be due to poor solubility. No abnormal clinical symptoms were noted following oral and intravenous administration of 97/63 in male rats. Rats showed normal activities same as untreated rats during sampling period.

## 4. Discussion

Compound analysis by HPLC-UV method is a traditional technique for quantification and quantitation as well as easily available at affordable cost. A well developed and validated HPLC-UV method is more effective as compared to LC-MS method which is a costly affair. So various parameters responsible for development of a new assay to quantify drugs by HPLC were optimized. The chromatographic conditions such as molarity and pH of buffer and type of columns were optimized. The results suggested that the bioanalytical method is accurate, as the bias is within the acceptance limits of ±20% of the theoretical value at LLOQ and ±15% at all other concentration levels. The precision around the mean value never exceeded 15% at any of the concentrations studied. Data from quality control samples revealed that the proposed method shows adequate specificity, sensitivity, accuracy, and precision.

No significant matrix suppression and/or enhancement as well as no endogenous peak interference were observed with peaks of interest. The absolute recoveries of 97/63 over the range 10–500 ng mL^−1^ as well as IS from serum were more than 74%. The result showed that the analytical method was accurate and precise over the linearity concentration range.

97/63 was subjected to long-term, F-T cycle, dry residual, reinjection stability studies and the compound showed good stability. No stability problems were encountered during storage and processing of pharmacokinetic samples.

Following oral and intravenous dose administration in male rats, the levels of 97/63 could be monitored in serum from the first dose sampling point, that is, 5 min. After oral administration 97/63 was rapidly absorbed from the gastrointestinal tract, attaining maximum serum level concentration (*C*_max⁡_) at 1 h after oral dose administration. A dominant *β* phase characterized much of the profile and exhibited clear log-linear behavior from ~6 to ~18 h after dose administration. The absorption rate constant (*K*_*a*_) was 0.82 h^−1^. No metabolite peaks were observed in rat serum. The observed rapid absorption of the compound after administering a single oral dose (72 mg kg^−1^) may also be due to the action of cosolvent DMSO which is known to have penetration enhancing property [18]. Rapid absorption of 97/63 will exert rapid onset of action against malarial parasite.

The low bioavailability may be due to first-pass metabolism of drug which might be excreted through the bile or variable rate of gastric emptying and motility of the gastrointestinal tract along the alimentary canal. Different degrees of drug response to the absorption site also influence the bioavailability. Metabolism in intestinal membrane during course of absorption as well as in liver is of major concern with the oral administration of many drugs, since it can reduce the systemic bioavailability of the drug. During gastrointestinal transit, the drug gets exposed to various pH conditions, gut flora, and enzymes due to which its systematic bioavailability may be reduced [19].

The low bioavailability of 97/63 can be improved by modifying it as produrg. Prodrug concepts at present are established tools for better fabrication of the physicochemical, biopharmaceutical, or pharmacokinetic properties of pharmacologically active agents [11]. Many times prodrug concept application at early stages of drug discovery program are fruitful rather than as a technique of last report due to high percentage of enhanced and successful uptake of drug by suitable prodrug formation [20, 21]. Thus, active drugs developed from prodrugs are considered as active metabolites. Prodrugs provide a rationale and opportunities to reach target physicochemical, pharmacokinetic, and pharmacodynamic properties. They can be designed to overcome pharmaceutical, pharmacokinetic, or pharmacodynamic barriers such as insufficient chemical stability, poor solubility, unacceptable taste or odor, irritation or pain, insufficient oral absorption, inadequate blood-brain barrier permeability, marked presystemic metabolism, and toxicity [22, 23]. With these concepts early stage development of prodrugs of 97/63 was undertaken and in due course of time different derivatives of 97/63 were synthesized. Among derivatised 97/63 compounds, prodrug 97/78 which was the hemisuccicinate derivative showed increased bioavailability of 97/63 manyfold [24].

## 5. Conclusion

The present bioanalytical assay was found to be specific, accurate, and precise over the linearity range of 10–500 ng mL^−1^. Liquid-liquid extraction method for sample preparation was efficient which shows extraction recovery of 97/63 from serum more than 74%. There were no stability problems for 97/63 during storage and sample processing hence fulfilling the criteria for bioanalytical methods. The relevance of pharmacokinetics in drug development has been well recognized and systematic interpretation of disposition behavior is of considerable use in reducing the expenditure and time involved in drug development besides optimization of drug therapy. Thus, a suitably validated method in different species can lead to rapid pharmacokinetic studies. This method will be applicable for further development and pharmacokinetic studies of 97/63. The observed low bioavailability (15.66%) and high variability of 97/63 can be attributed to lower log *P* value (<3.5) which indicates poor absorption of the compound in the aqueous medium and less lipophillic behavior and hence produrg 97/78 was synthesized as hemisuccinate derivative of 97/63 which enhanced the solubility and absorption manyfold [24].

## Figures and Tables

**Table 1:** Absolute recoveries of 97/63 in rat serum.

Spiked concentration (ng mL^−1^)	% absolute recovery (Mean ± SD, *n* = 3)
10	78.38 ± 4.24
25	87.78 ± 4.51
50	76.51 ± 0.02
100	76.14 ± 0.33
250	74.18 ± 0.29
500	74.72 ± 0.05

**Table 2:** Accuracy and precision of 97/63 in rat serum (*N* = 5).

Spiked Concentration (ng mL^−1^)	Accuracy (% bias)		Precision (% RSD)	
	Interday	Intraday	Interday	Intraday
10	−14.00	−0.70	14.47	14.8
100	9.64	6.83	14.06	5.56
500	13.42	6.83	5.74	4.71

**Table 3:** Pharmacokinetic parameter of 97/63 after oral administration at 72 mg kg^−1^ and intravenous administration at 18 mg kg^−1^ dose of 97/63 in male rats (mean ± SD, *N* = 4).

PK parameter	Oral	Intravenous
*C*_0_ (ng mL^−1^)	∗	1799.99 ± 330.24
*C*_max⁡_ (ng mL^−1^)	229.24 ± 64.26	∗
*T*_max⁡_ (h)	1.00 ± 0.7	∗
*T*_1/2_ (α) (h)	0.84 ± 0.14	0.45 ± 0.08
*T*_1/2_ (*β*) (h)	10.61 ± 0.2	10.57 ± 0.16
AUC_0–*∞*_ (ng h mL^−1^)	1268.97 ± 27.04	2025.75 ± 574.3
Vd (L kg^−1^)	172.31 ± 28.59	134.7 ± 11.17
Cl (L h^−1^ Kg^−1^)	11.2 ± 0.07	8.89 ± 0.11
% bioavailability	15.66	—

*Not applicable.
